# Elucidating the Role of K^+^ Channels during In Vitro Capacitation of Boar Spermatozoa: Do SLO1 Channels Play a Crucial Role?

**DOI:** 10.3390/ijms20246330

**Published:** 2019-12-15

**Authors:** Marc Yeste, Marc Llavanera, Guillermo Pérez, Fabiana Scornik, Josep Puig-Parri, Ramon Brugada, Sergi Bonet, Elisabeth Pinart

**Affiliations:** 1 Unit of Cell Biology, Biotechnology of Animal and Human Reproduction (TechnoSperm), Department of Biology, Faculty of Sciences, Institute of Food and Agricultural Technology, University of Girona, E-17003 Girona, Spain; marc.yeste@udg.edu (M.Y.); marc.llavanera@udg.edu (M.L.); joseppuig11@gmail.com (J.P.-P.); sergi.bonet@udg.edu (S.B.); 2 Department of Medical Sciences, Faculty of Medicine, University of Girona, E-17003 Girona, Spain; gperez@gencardio.com (G.P.); fscornik@gencardio.com (F.S.); rbrugada@gencardio.com (R.B.); 3 Cardiovascular Genetics Group, Girona Biomedical Research Institute (IDIBGI), E-17190 Girona, Spain; 4 Biomedical Research Networking Center on Cardiovascular Diseases (CIBERCV), E-28029 Madrid, Spain; 5 Cardiology Service, Hospital Josep Trueta, E-17003 Girona, Spain

**Keywords:** in vitro capacitation, boar spermatozoa, SLO1 channels, PAX, TEA

## Abstract

This study sought to identify and localize SLO1 channels in boar spermatozoa by immunoblotting and immunofluorescence, and to determine their physiological role during in vitro sperm capacitation. Sperm samples from 14 boars were incubated in a capacitation medium for 300 min in the presence of paxilline (PAX), a specific SLO1-channel blocker, added either at 0 min or after 240 min of incubation. Negative controls were incubated in capacitation medium, and positive controls in capacitation medium plus tetraethyl ammonium (TEA), a general K^+^-channel blocker, also added at 0 min or after 240 min of incubation. In all samples, acrosome exocytosis was triggered with progesterone after 240 min of incubation. Sperm motility and kinematics, integrity of plasma and acrosome membranes, membrane lipid disorder, intracellular calcium levels and acrosin activity were evaluated after 0, 60, 120, 180, 240, 250, 270 and 300 min of incubation. In boar spermatozoa, SLO1 channels were found to have 80 kDa and be localized in the anterior postacrosomal region and the mid and principal piece of the tail; their specific blockage through PAX resulted in altered calcium levels and acrosome exocytosis. As expected, TEA blocker impaired in vitro sperm capacitation, by altering sperm motility and kinematics and calcium levels. In conclusion, SLO1 channels are crucial for the acrosome exocytosis induced by progesterone in in vitro capacitated boar spermatozoa.

## 1. Introduction

Capacitation includes a set of physiological changes that are required for a spermatozoon to be able to fertilize the oocyte. These changes include augmented beat frequency and curvilinear motility, increase in intracellular pH, rise in intracellular calcium levels, plasma membrane hyperpolarization and tyrosine phosphorylation of certain sperm proteins (reviewed in [1,2,3]). Ion channels orchestrate such a sequence of events, despite differences existing between mammalian species not only in nature, but also in their regulation mechanisms [4,5,6,7]. For instance, voltage-gated H^+^ channel 1 (HVCN1) is functional in human but not in mouse spermatozoa [4]; in contrast, purinergic P2X channels are functional in mouse [5], but not in human spermatozoa [7]. CatSper, a sperm-specific pH-sensitive, voltage-gated Ca^2+^ channel is activated by progesterone in humans, but not in mice [8,9].

While mounting evidence supports the crucial role of potassium (K^+^) channels in somatic cells, little data exist about the localization and regulation of these channels in mammalian spermatozoa [10]. Previous studies have reported that SLO channels are the main K^+^ channels in human and mouse spermatozoa. These channels, which belong to the *SLO* gene family [2,3,9,11,12], have been reported to play a vital role in the regulation of sperm volume, and have also been found to be involved in the sequence of changes that take place during capacitation [2,9,13]. Remarkably, whereas such a regulating role is mainly performed by SLO3 in mouse [1,11,12,14], it involves both SLO1 [2] and SLO3 [3] in human spermatozoa. SLO1 channels or big potassium (BK) or maxi K^+^ channels, exhibit high sensitivity to changes of both voltage and intracellular Ca^2+^ levels [15,16], whereas SLO3 channels are highly sensitive to intracellular alkalinization [9].

Given the already reported differences between human and mouse spermatozoa, further research involving other species (such as pig) is much warranted. For this reason, this study sought to determine the presence and localization of SLO1 channels in boar spermatozoa, and to investigate whether they play any role during in vitro capacitation. This functional approach was performed pharmacologically by using paxilline (PAX), a specific SLO1-channel blocker. Paxilline is a fungal indole alkaloid that acts as a potent and specific inhibitor of SLO1 channels [2,15,17,18]; its inhibitory role is produced by stabilizing the close-channel conformation rather than by occluding the open-channel [19]. As positive control, we incubated sperm samples with tetraethyl ammonium chloride (TEA), a quaternary ammonium compound with a broad inhibiting effect on several K^+^ transporters [19,20]; its inhibiting mechanism is due to the entrance of the TEA molecule into the pore, blocking both closed and open channels [15]. To ensure that nearly all K^+^-channels were blocked, TEA was used at a high concentration (20 mM). 

## 2. Results

To understand the physiological role of SLO1 channels during in vitro capacitation, boar spermatozoa were incubated in a capacitation medium for 300 min in the presence of PAX or TEA blockers. Two sets of experiments were devised. In Experiment 1, PAX (PAX samples) or TEA (TEA samples) were added to the capacitation medium at time 0, whereas in Experiment 2, PAX (PAX acute samples) or TEA (TEA acute samples) were added at 240 min of incubation. In all trials, progesterone was added at 240 min of incubation.

### 2.1. Identification and Immunolocalization of SLO1 Channels

**Figure 1:**
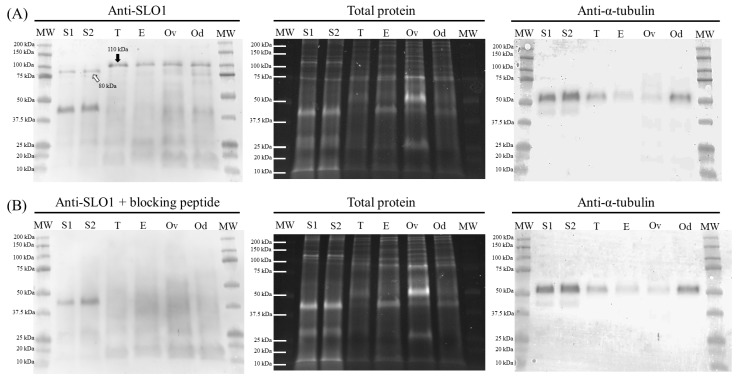
Representative immunoblot of sperm (S1 and S2) and tissue (positive controls; testicle [T], epididymis [E], ovary [Ov] and oviduct [Od]) samples for SLO1 channels (**A**), and its corresponding peptide competition assay (**B**). TGX™ (Tris-glycine extended) Stain Free (total protein) and α-tubulin (anti-α-tubulin) were performed as loading controls.

**Figure 2:**
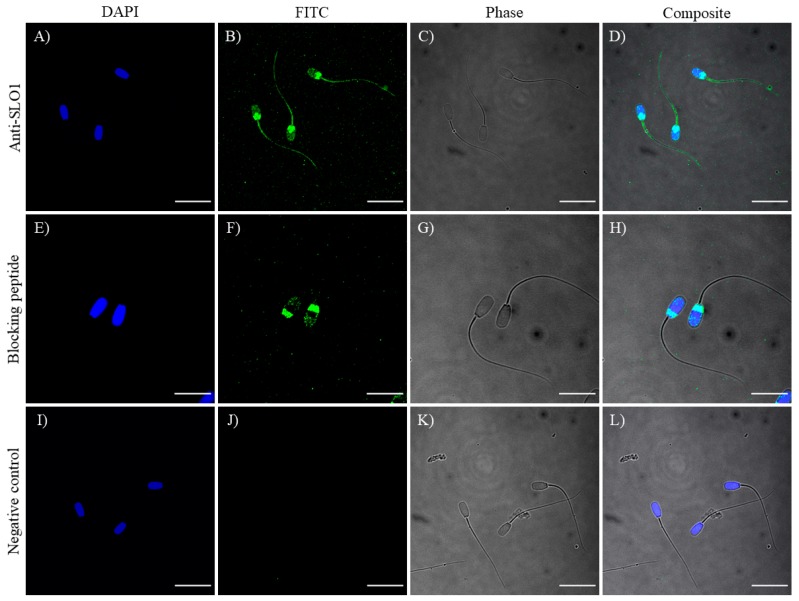
Immunolocalization of SLO1 (**A**–**D**), with its corresponding peptide competition assay (**E**–**H**) and negative control (**I**–**L**). Nucleus is shown in blue (DAPI; 4′,6-diamidino-2-phenylindole) and SLO1 is shown in green (FITC; Fluorescein isothiocyanate). Phase images of sperm cells were collected using a transmitted light detector (Phase). Scale bars: (**A**–**D**) and (**I**–**L**): 20 μm; (**E**–**H**): 13.5 μm.

Immunoblotting assays confirmed the presence of a SLO1-specific band of 80 kDa in ejaculated sperm samples. As positive controls, samples from boar testis, epididymis, ovary and oviduct showed a single 110 kDa-band corresponding to SLO1 channels. Peptide competition assays proved that the 80 kDa-band appearing in sperm samples was specific of SLO1 (Figure 1). On the other hand, immunofluorescence studies exhibited a labeling in the acrosomal region and the anterior and posterior areas of the postacrosomal region, as well as in the mid-piece, principal piece and terminal piece. Peptide competition assays resulted in the total loss of staining affinity to the anterior post-acrosomal region and to the sperm flagellum, thus, indicating the specific localization of SLO1 channels in these regions (Figure 2). In contrast, the acrosomal and posterior post-acrosomal regions did not change their staining in the presence of the blocking peptide, which would agree with the unspecific 40 kDa band observed in immunoblotting assays.

### 2.2. Sperm Motility

#### 2.2.1. Total and Progressive Motility and Hypermotility

**Figure 3:**
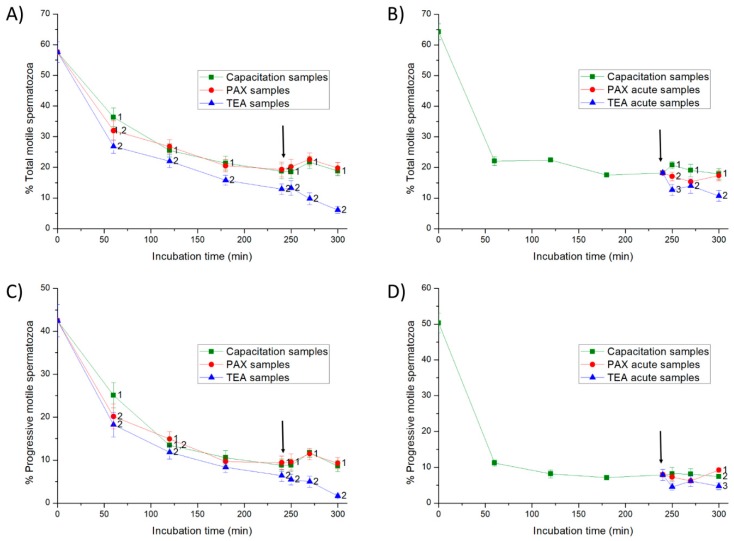
Percentages of total and progressive motile spermatozoa throughout in vitro incubation of sperm in capacitation medium (negative control samples), capacitation medium plus TEA (tetraethyl ammonium) blocker (positive control samples), and capacitation medium plus PAX (paxilline) blocker. In experiment 1, TEA (TEA samples) or PAX (PAX samples) blockers were added to the capacitation medium at 0 min (**A**,**C**). In experiment 2, TEA (TEA acute samples) or PAX (PAX acute samples) blockers were added to the capacitation medium at 240 min of incubation (**B**,**D**). Different superscripts indicate significant differences (*p* < 0.05) between samples within the same time point. The arrow indicates the time at which 10 µg/mL of progesterone was added in all samples (i.e., 240 min of incubation).

**Figure 4:**
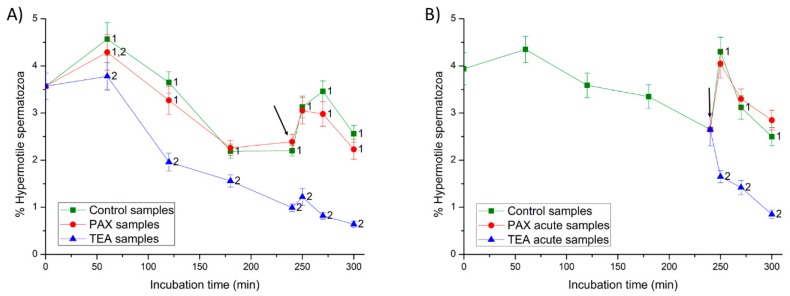
Percentages of hypermotile spermatozoa (i.e., hyperactive spermatozoa with amplitude of lateral head displacement (ALH) > 2.5 µm and straightness (STR) > 85%) throughout in vitro incubation of sperm in capacitation medium (negative control samples), capacitation medium plus TEA blocker (positive control samples), and capacitation medium plus PAX blocker. In experiment 1, TEA (TEA samples) or PAX (PAX samples) blockers were added to the capacitation medium at 0 min (**A**). In experiment 2, TEA (TEA acute samples) or PAX (PAX acute samples) blockers were added to the capacitation medium at 240 min of incubation (**B**). Different superscripts indicate significant differences (*p* < 0.05) between samples within the same time point. The arrow indicates the time at which 10 µg/mL of progesterone was added in all samples (i.e., 240 min of incubation).

In Experiment 1, the addition of progesterone at 240 min in control and PAX samples had little effect on total and progressive motilities from 250 to 300 min (*p* > 0.05) (Figure 3), but it significantly increased the percentages of hypermotile spermatozoa (i.e., hyperactive spermatozoa with ALH > 2.5 µm and STR > 85%; *p* < 0.05; Figure 4). In TEA samples (positive control) the addition of progesterone did not result in significant variations in total and progressive motilities from 250 to 270 min of incubation, but they decreased significantly (*p* < 0.05) at 300 min; hypermotility, however, increased transiently and non-significantly (*p* > 0.05) at 250 min of incubation.

In Experiment 2, the addition of progesterone to the capacitation medium (negative control) and the joint addition of progesterone and PAX (PAX acute samples) had little effect on sperm motility (*p* > 0.05) (Figure 3), but they led to a peak in the percentage of hypermotile spermatozoa at 250 min of incubation (*p* < 0.05) (Figure 4). In contrast, the addition of progesterone and TEA (TEA acute samples, positive control) resulted in a significant (*p* < 0.05) decrease of total and progressive motilities at 250 min of incubation, and of hypermotility from 250 to 300 min of incubation (*p* < 0.05).

#### 2.2.2. Curvilinear (VCL), Straight-Line (VSL) and Average Pathway (VAP) Velocities

In Experiment 1, the addition of progesterone to negative control samples did not result in significant variations in VCL, VSL and VAP parameters (*p* > 0.05) (Supplementary Table S1). In PAX samples, the addition of progesterone led to a significant (*p* < 0.05) decrease in VCL at 300 min; VSL significantly (*p* < 0.05) increased at 270 min and then decreased at 300 min. In TEA samples (positive control), all velocity parameters decreased progressively throughout the experiment, even following the addition of progesterone.

In Experiment 2, the addition of progesterone (negative control samples) and the joint addition of progesterone and PAX (PAX acute samples) at 240 min of incubation resulted in a significant (*p* < 0.05) increase of VCL at both 250 and 300 min, and in a significant (*p* < 0.05) increase in VSL and VAP at 300 min. In contrast, the joint addition of progesterone and TEA (TEA acute samples, positive control) led to a significant (*p* < 0.05) decrease in all velocity parameters at 250 min of incubation.

#### 2.2.3. Indexes of Linearity (LIN), Straightness (STR) and Oscillation (WOB)

In Experiment 1, the addition of progesterone at 240 min resulted in a significant (*p* < 0.05) increase of LIN and STR indexes at 270 min in negative controls and PAX samples, but it did not result in significant variations in TEA samples (Supplementary Table S1).

In Experiment 2, the addition of progesterone to negative control samples at 240 min resulted in a significant (*p* < 0.05) increase of LIN and STR at 300 min of incubation, and a significant (*p* < 0.05) increase of WOB at 270 min. The joint addition of progesterone and PAX (PAX acute samples) led to a significant (*p* < 0.05) increase in these three indexes at 270 min. Finally, the joint addition of progesterone and TEA (TEA acute samples, positive control) significantly increased (*p* < 0.05) LIN at 300 min and STR at 270 min of incubation.

#### 2.2.4. Amplitude of Lateral Head Displacement (ALH) and Beat Cross Frequency (BCF)

In Experiment 1, the addition of progesterone to negative control samples led to a significant (*p* < 0.05) decrease of ALH at 250 min, whereas in PAX samples it did not have any significant effect. In TEA samples (positive control), ALH and BCF significantly (*p* < 0.05) decreased throughout all the experimental period (Supplementary Table S1).

In Experiment 2, the addition of progesterone to negative control samples resulted in a significant (*p* < 0.05) increase in both ALH and BCF at 300 min of incubation. The joint addition of progesterone and PAX (PAX acute samples) led to a significant (*p* < 0.05) increase in ALH at 300 min. Finally, the joint addition of progesterone and TEA (TEA acute samples, positive control) did not have any effect on either ALH or BCF.

### 2.3. Plasma and Acrosome Membrane Integrities

**Figure 5:**
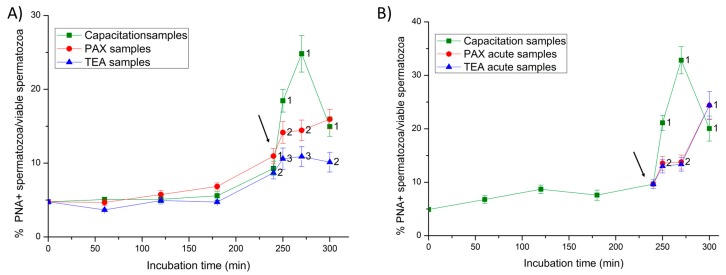
Percentages of viable spermatozoa with exocytosed acrosome (PNA-) in relation to total viable spermatozoa (EthD-1-) throughout in vitro incubation of sperm samples in capacitation medium (negative control samples), capacitation medium plus TEA blocker (positive control samples), and capacitation medium plus PAX blocker. In Experiment 1, TEA (TEA samples) or PAX (PAX samples) blockers were added to the capacitation medium at 0 min (**A**). In Experiment 2, TEA (TEA acute samples) or PAX (PAX acute samples) blockers were added to the capacitation medium at 240 min of incubation (**B**). Different superscripts indicate significant differences (*p* < 0.05) between samples within the same time point. The arrow indicates the time at which 10 µg/mL of progesterone was added in all samples (i.e., 240 min of incubation).

In the first 240 min of incubation, less than 10% of viable spermatozoa showed an exocytosed acrosome in both Experiments 1 and 2 (Figure 5). In Experiment 1, the addition of progesterone led to a significant increase (*p* < 0.05) in the percentage of viable spermatozoa with an exocytosed acrosome at 250 min. Nevertheless, the increase was significantly higher (*p* < 0.05) in negative control samples, and significantly lower (*p* < 0.05) in positive control (TEA) samples.

In Experiment 2, the addition of progesterone to control negative samples led to a fast and significant (*p* < 0.05) increase in the percentage of viable spermatozoa with an exocytosed acrosome from 250 min of incubation. In contrast, the joint addition of progesterone and PAX (PAX acute samples), and that of progesterone and TEA (TEA acute samples, positive control) resulted in a delayed increase of that percentage at 300 min of incubation.

### 2.4. Membrane Lipid Disorder

**Figure 6:**
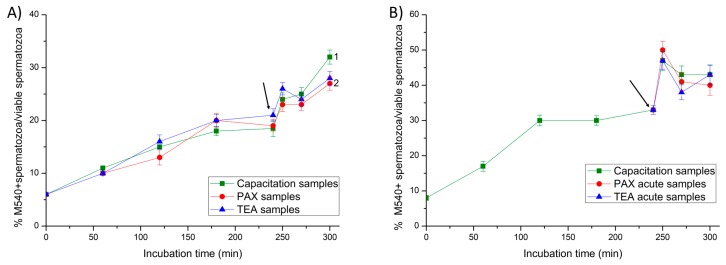
Percentages of viable spermatozoa with high lipid disorder (M540+) in relation to total viable spermatozoa (YO-PRO-1-) throughout in vitro incubation of sperm samples in capacitation medium (negative control samples), capacitation medium plus TEA blocker (positive control samples), and capacitation medium plus PAX blocker. In Experiment 1, TEA (TEA samples) or PAX (PAX samples) blockers were added to the capacitation medium at 0 min (**A**). In Experiment 2, TEA (TEA acute samples) or PAX (PAX acute samples) blockers were added to the capacitation medium at 240 min of incubation (**B**). Different superscripts indicate significant differences (*p* < 0.05) between samples within the same time point. The arrow indicates the time at which 10 µg/mL of progesterone was added in all samples (i.e., 240 min of incubation).

In all samples, membrane lipid disorder of viable spermatozoa increased progressively during the first 240 min of incubation (Figure 6). In Experiment 1, the addition of progesterone resulted in a significant peak (*p* < 0.05) of the percentage of viable spermatozoa with high membrane lipid disorder at 250 min of incubation in negative and positive (TEA) control samples, and in PAX samples.

Similarly, in Experiment 2, the addition of progesterone (negative control samples), the joint addition of progesterone and PAX (PAX acute samples), and that of progesterone and TEA (TEA acute samples, positive control) also resulted in a significant (*p* < 0.05) increase in the percentage of viable spermatozoa with high membrane lipid disorder at 250 min.

### 2.5. Intracellular Calcium Levels

**Figure 7:**
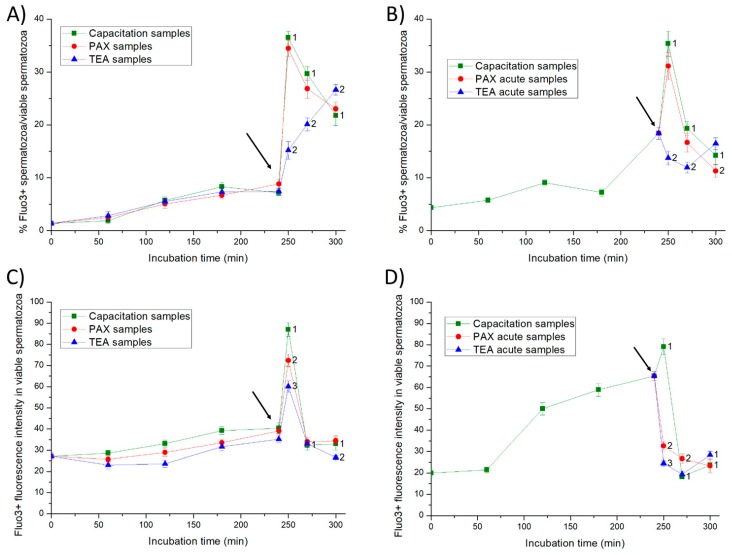
Percentages of viable spermatozoa with high intracellular calcium levels (Fluo3+) in relation to total viable spermatozoa (PI-) (**A**,**B**) and fluorescence intensity of Fluo3+ in viable spermatozoa with positive Fluo3-staining (**C**,**D**) throughout in vitro incubation of sperm samples in capacitation medium (negative control samples), capacitation medium plus TEA blocker (positive control samples), and capacitation medium plus PAX blocker. In experiment 1, TEA (TEA samples) or PAX (PAX samples) blockers were added to the capacitation medium at 0 min (**A**,**C**). In experiment 2, TEA (TEA acute samples) or PAX (PAX acute samples) blockers were added to the capacitation medium at 240 min of incubation (**B**,**D**). Different superscripts indicate significant differences (*p* < 0.05) between samples within the same time point. The arrow indicates the time at which 10 µg/mL of progesterone was added in all samples (i.e., 240 min of incubation).

In Experiment 1, less than 10% of viable spermatozoa showed positive Fluo3-staining (Fluo3+) during the first 240 min of incubation in all samples (Figure 7). The addition of progesterone resulted in a significant peak (*p* < 0.05) of the percentage of viable spermatozoa showing Fluo3+ staining at 250 min of incubation. Such an increase was significantly higher (*p* < 0.05) in both negative control and PAX samples than in positive control (TEA) samples. While in all samples, the fluorescence intensity of Fluo3 in viable sperm cells with positive Fluo3-staining reached a significant peak (*p* < 0.05) at 250 min of incubation, the extent of that peak differed between treatments (Figure 7). In effect, in comparison to PAX samples, the peak was significantly higher (*p* < 0.05) in negative control samples and significantly lower (*p* < 0.05) in positive control (TEA) samples.

In Experiment 2, the addition of progesterone to negative control samples, and the joint addition of progesterone and PAX (PAX acute samples) led to a significant (*p* < 0.05) peak in the percentage of viable spermatozoa with positive Fluo3-staining at 250 min of incubation. In contrast, the joint addition of progesterone and TEA (TEA acute samples, positive control) had no effect on this parameter. In both negative control samples and PAX acute samples, the fluorescence intensity of Fluo3 in viable spermatozoa with positive Fluo3-staining reached a significant (*p* < 0.05) peak at 250 min of incubation. Such a significant increase in the fluorescence intensity was not observed in TEA acute samples (positive control).

**Figure 8:**
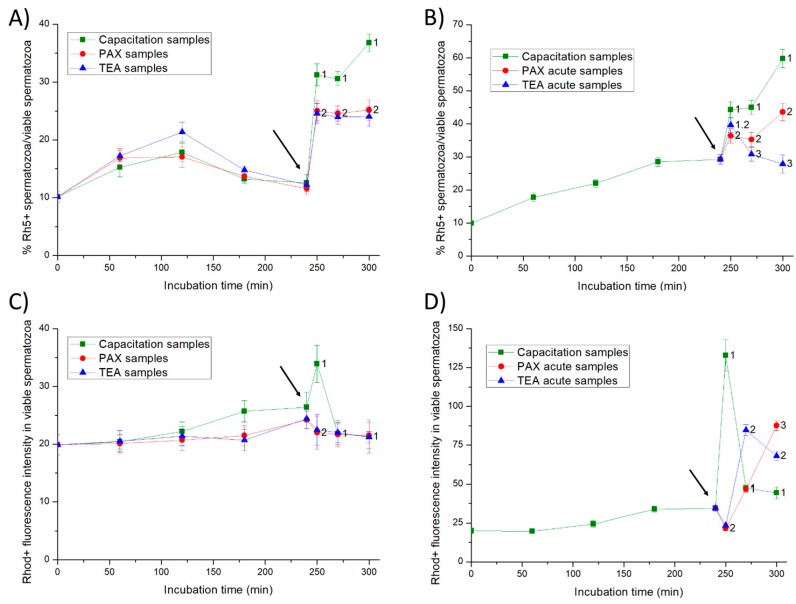
Percentages of viable spermatozoa with high calcium levels evaluated through Rhod5 (Rh5+) in relation to total viable spermatozoa (YO-PRO-1-) and fluorescence intensity of Rhod5 in viable spermatozoa with positive Rhod5-staining (**C**,**D**) throughout in vitro incubation of sperm samples in capacitation medium (negative control samples), capacitation medium plus TEA blocker (positive control samples), and capacitation medium plus PAX blocker. In experiment 1, TEA (TEA samples) or PAX (PAX samples) blockers were added to the capacitation medium at 0 min (**A**,**C**). In experiment 2, TEA (TEA acute samples) or PAX (PAX acute samples) blockers were added to the capacitation medium at 240 min of incubation (**B**,**D**). Different superscripts indicate significant differences (*p* < 0.05) between samples within the same time point. The arrow indicates the time at which 10 µg/mL of progesterone was added in all samples (i.e., 240 min of incubation).

As far as intracellular calcium levels evaluated by Rhod5 are concerned (Figure 8), the addition of progesterone resulted in a significant (*p* < 0.05) increase in the percentage of viable spermatozoa with positive Rhod5-staining from 250 to 300 min of incubation in all samples. Nevertheless, such an increase was significantly higher (*p* < 0.05) in negative control samples than in both PAX and TEA (control positive) samples. In negative control samples, the fluorescence intensity of Rhod5 in viable spermatozoa with positive Rhod5-staining reached a significant (*p* < 0.05) increase at 250 min of incubation (Figure 8). In contrast, such a peak was not observed in either PAX or TEA acute (negative control) samples.

In Experiment 2, changes in the percentages of viable spermatozoa with Rhod5-positive staining differed significantly (*p* < 0.05) between samples. Therefore, the addition of progesterone to negative control samples and the joint addition of progesterone and PAX (PAX acute samples) led to a significant (*p* < 0.05) increase in the percentages of viable spermatozoa with positive Rhod-staining from 250 to 300 min of incubation. Nevertheless, such a decrease was less apparent in PAX acute than in negative control samples. The joint addition of progesterone and TEA (TEA acute samples, positive control) resulted in a transient but significant (*p* < 0.05) increase in the percentage of viable spermatozoa with positive Rhod5-staining at 250 min of incubation. In negative control samples, the fluorescence intensity of Rhod5 in viable spermatozoa with positive Rhod5-staining also increased significantly (*p* < 0.05) from 250 min to 300 of incubation. As compared to negative control samples, PAX and TEA acute (negative control) samples showed a delayed and less apparent increase in the fluorescence intensity of Rhod5.

### 2.6. Acrosin Activity

**Figure 9:**
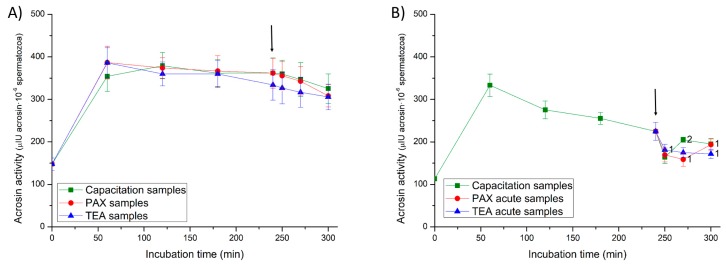
Acrosin activity (µIU/10^6^ spermatozoa) throughout in vitro incubation of sperm samples in capacitation medium (negative control samples), capacitation medium plus TEA blocker (positive control samples), and capacitation medium plus PAX blocker. In experiment 1, TEA (TEA samples) or PAX (PAX samples) blockers were added to the capacitation medium at 0 min (**A**). In experiment 2, TEA (TEA acute samples) or PAX (PAX acute samples) blockers were added to the capacitation medium at 240 min of incubation (**B**). Different superscripts indicate significant differences (*p* < 0.05) between samples within the same time point. The arrow indicates the time at which 10 µg/mL of progesterone was added in all samples (i.e., 240 min of incubation).

In Experiment 1, the addition of progesterone at 240 min of incubation did not have any significant effect on acrosin activity, whatever the sample type. In Experiment 2, the addition of progesterone and the joint addition of progesterone and PAX resulted in a transient but significant (*p* < 0.05) increase in the enzyme activity in negative control and PAX samples (Figure 9). In contrast, the joint addition of progesterone and TEA led to a significant (*p* < 0.05) decrease in acrosin activity in TEA acute samples (positive control) at 250 min of incubation.

## 3. Discussion

Although accumulating evidence supports the relevance of ion channels in mammal sperm physiology, most studies are performed in human and mouse spermatozoa [2,3,9,10,15], and little data exist on their presence, localization and role in other mammal species. In the present study, we have identified and localized SLO1 channels in boar spermatozoa by immunoblotting and immunofluorescence. We have also analyzed pharmacologically their physiological role during in vitro capacitation by using paxilline as a specific blocker of this channel. It is worth mentioning that this blocker has already been used at a similar concentration to that used in our approach [2,15,21].

Our data show, for the first time, a specific 80 kDa-band corresponding to SLO1 channels in boar spermatozoa. An interesting finding of our study is that the molecular weight of SLO1 channels in boar spermatozoa is lower than in other tissues, such as the testis, epididymis, ovary and oviduct (MW: 110 kDa). Although molecular heterogeneity of SLO1 channels has been reported in several species and tissues, the specific composition of SLO1 channels remains controversial (reviewed in [22]). SLO1 channels contain four pore-forming subunits, which are encoded by a single *SLO1* gene [23]. The wide diversity of mature transcripts could be explained by the alternative splicing to which *SLO1*-mRNA is subjected [24]. Each SLO1 channel has three structural domains: the voltage-sensing domain (VSD), the cytosolic domain and the pore-gate domain (PGD; reviewed by [25,26]). The voltage-sensing domain is sensitive to membrane potential, whereas the cytosolic domain has high affinity Ca^2+^-binding sites and confers high sensibility to changes in both voltage and intracellular Ca^2+^ concentration. The pore-gate domain (PGD) regulates K^+^-conductance by opening or closing the channel [25,26]. In this context, it is worth mentioning that alternative mRNA splicing provides different functional properties to SLO1 channels, since it results in modified calcium and voltage sensitivity, and in changes in its expression at the cell surface level (reviewed in [27,28]). In several tissues, SLO1 heterogeneity has been correlated with different sensitivities and biophysical responses [22]. Therefore, the results observed in this study suggest the presence of a specific SLO1 variant in the plasma membrane of boar spermatozoa, in agreement with the structural and functional diversity of this channel. Since the identification of such sperm specific SLO1 variant is out of the scope of the present study, further investigations aimed at identifying the splice variant/s of SLO1 channels present in boar spermatozoa are warranted.

The present study shows that SLO1 channels are widely distributed throughout the sperm flagellum. Interestingly, SLO1 is also present in the anterior area of the post-acrosomal region of boar spermatozoa. The localization of SLO1 channels in boar spermatozoa appear to differ from that described in human spermatozoa, in which SLO1 channels distribute throughout the principal piece [2]. To the best of our knowledge, no data exist about the distribution of SLO1 channels in the sperm of other mammalian species. The differences in the localization of SLO1 channels between human and boar spermatozoa highlight the divergences between species with regard to the regulation mechanisms of their physiology. In human sperm, both SLO1 channels [2] and CatSper channels [3,9] are localized in the principal piece. Such a physical proximity has been related to the functional relationship between both types of channels [10]. Since SLO1 channels are Ca^2+^-sensitive [29], the close proximity to CatSper channels suggests that K^+^ conductance is directly dependent on calcium entrance in human spermatozoa. In contrast, in boars, such a close proximity between SLO1 and CatSper channels is less apparent, since in this species, CatSper channels localize in the principal piece [30] and SLO1 channels distribute throughout the sperm flagellum and the anterior post-acrosomal region. Taking into account that the vesicular membranous structures of the post-acrosomal region act as calcium stores (reviewed in [31]), we can hypothesize that, in boar spermatozoa, the presence of SLO1 channels in the anterior area of the post-acrosomal region could be related to the internal mobilization of Ca^2+^ reservoirs in this sperm region.

Sperm incubation in the capacitation medium resulted in a reduction in the percentage of both viable and motile spermatozoa during the first 120 min of incubation. Similar decreases were also reported in in vitro capacitation of boar spermatozoa [32,33]. In contrast, acrosin activity increased 2.5-fold in the first 60 min of incubation, reaching a value that remained nearly constant until the end of the experiment, in accordance with previous results [32,34]. Acrosin is a tripsin-like proteinase present in epididymal and ejaculated spermatozoa as an inactive zymogen (proacrosin) [35], which distributes uniformly over the sperm head [32]. During in vitro capacitation, proacrosin is converted into acrosin by proteolitic cleavage, and acrosin redistributes towards the apical ridge to further undergo acrosome reaction [32]. In agreement with previous reports, the addition of progesterone induces increased membrane lipid disorder and intracellular calcium levels in the sperm head, both events being crucial for acrosome exocytosis. Progesterone also rises intracellular calcium levels in the flagellum, which are closely related with changes in sperm kinematics, especially in linearity, straightness and lateral head displacement [32,33,36,37,38]. 

As expected, TEA blocker impairs in vitro capacitation of boar spermatozoa by reducing sperm motility, hypermotility and kinematics, acrosome integrity, and intracellular calcium levels. These results highlight the close relationship between Ca^2+^ and K^+^ conductance during sperm capacitation. However, K^+^ conductance does not seem to be related with changes in membrane lipid disorder, as demonstrated by the lack of differences between control samples and PAX and TEA samples. Interestingly, SLO1 blocking does not induce significant alterations in sperm motility during in vitro capacitation, but it leads to an impaired acrosome exocytosis after progesterone addition. An interesting finding of our work is that whereas TEA and PAX blockers showed similar effects on the intracellular calcium levels marked by Rhod5, which has more affinity for the calcium stored in the sperm head [33], they had a different impact on the levels of intracellular calcium residing in the mid-piece and stained by Fluo3. This impact upon the calcium levels of the sperm head, preferentially marked by Rhod5, was concomitant with progesterone-induced acrosome exocytosis. Remarkably, the lack of differences between TEA and PAX blockers strongly suggests, for the first time, that SLO1 channels have an essential role in triggering calcium conductance in the sperm head during in vitro capacitation of boar spermatozoa and are involved in the acrosome exocytosis induced by progesterone. Our results also indicate that the rises in intracellular calcium levels in the sperm head and tail induced by progesterone are not related, in accordance with previous reported data in human [39] and mouse [40] spermatozoa. Lack of alterations in the intracellular calcium levels marked by Fluo3 after SLO1-inhibition suggests that not only SLO1 channels, but also other K^+^ channels are involved in K^+^ conductance in the flagellum. Thus, further research is needed to identify which K^+^ channels are implicated in the K^+^ conductance across boar sperm tail.

The inhibitory effect of PAX was higher when added at 0 min than when added at 240 min. According to the blocking mechanism described by Zhou et al. [18], PAX toxin does not induce the occlusion of opened-SLO1 channels, but it stabilizes the closed-channel conformation. Taking this into account and given the effects of PAX when added at 0 min, one could suggest that the number of SLO1 channels in the closed conformation increases progressively throughout the incubation period. In contrast, the reduced inhibitory effect when PAX was added at 240 min indicates that only few SLO1 channels are in the closed conformation. We can also hypothesize that the ability of PAX to inhibit SLO1 channels could be reduced when added together with progesterone. Progesterone is known to induce a fast rise in intracellular calcium levels upon its addition [33] and intracellular calcium regulates the opened-closed state of SLO1 channels [41]. In effect, each SLO1 channels have calcium binding sites in the cytoplasmic domain and the calcium binding to such sites induces a conformational change that opens SLO1 [41]. SLO1 channels are also regulated by membrane potential; membrane hyperpolarization modifies the conformation of the voltage-sensing domain leading to the opened state and increasing calcium-affinity of the binding sites [41].

Our results show that TEA and PAX differ in their inhibitory effect on the sperm tail. Therefore, whereas TEA inhibits both sperm motility and the capacitation-induced increase of Fluo3-marked calcium in the mid-piece, either when added at 0 min or at 240 min of incubation, the impact of PAX is less apparent. The observed effects of TEA match with its high capacity to block K^+^-channels, regardless of whether they are opened or closed [15]. With regard to the decrease in sperm motility, we also found a significant decrease in sperm velocity parameters (VCL, VSL and VAP) and in the beat cross frequency (BCF). In agreement with these findings, non-selective blocking of K^+^ channels have also been found to inhibit progressive motility in human spermatozoa [11,12]. Furthermore, our data also highlight the importance of K^+^ conductance for sperm motility during in vitro capacitation, as previously reported in mouse [13]. However, the lack of an apparent effect of PAX on sperm motility during in vitro capacitation strongly suggests that, in boar spermatozoa, K^+^ conductance across the flagellum is performed by different types of K^+^ channels. In this context, it is worth mentioning that the actual role of SLO1 channels in sperm physiology is species-specific, as while they are involved in the regulation of human sperm motility, they do not play such a role in mouse spermatozoa [2,3]. These divergences suggest that differences exist in the content and physiological regulation of K^+^ channels exist between species. 

## 4. Materials and Methods

### 4.1. Materials

All chemicals were purchased from Sigma-Aldrich Química (Madrid, Spain) unless otherwise indicated.

### 4.2. Semen and Tissue Samples

The present study was performed using commercial seminal doses from 14 Piétrain boars, which were kept under adjusted conditions of temperature and humidity, fed a standard diet, and provided with water ad libitum. The semen collection rhythm was twice a week, and the boar stud recorded no fertility problems for any of the males included in these experiments. Ejaculates were collected using the glove-hand technique, and the sperm-rich fraction was immediately filtered to remove the gel, pre-diluted 2:1 (*v*/*v*) in a long-term extender (Vitasem, Magapor, Ejea de los Caballeros, Zaragoza, Spain) at 37 °C inside a collecting recipient, and packed into 90 mL commercial doses at a concentration of 3 × 10^9^ spermatozoa/dose. Commercial seminal doses were then cooled down to 16 °C, and three doses per collection and boar were sent to our laboratory in a heat-insulating recipient at 16 °C. Once in the lab, sperm quality was checked by assessing motility and morphology using a computer assisted sperm analysis (CASA) system (see Section 4.6), and viability. Sperm viability was assessed through SYBR14/PI staining (LIVE/DEAD Sperm Viability Kit; Molecular Probes, Invitrogen, ThermoFisher Scientific; Waltham, MA, USA) and flow cytometry (see Section 4.7) [42]. All seminal doses used in the present study had a sperm quality above the following thresholds: 70% of total motile spermatozoa, 80% of viable spermatozoa and 85% of morphologically normal spermatozoa (data not shown).

Tissue samples (testicles, epididymis, ovarian and oviduct) for Western blot analysis were obtained from healthy, sexually mature boars at a local slaughterhouse (Frigoríficos Costa Brava; Riudellots de la Selva, Spain).

### 4.3. Experimental Design

For each boar, a first approach was performed to detect the presence of SLO1 channels in boar spermatozoa by immunoblotting and their localization through immunofluorescence.

Upon confirmation of the presence of SLO1 channels in boar spermatozoa, we designed two sets of experiments to analyze the effects of adding either PAX or TEA to the capacitation medium during in vitro capacitation. Each experiment included seven boars.

In the first experiment, two seminal doses of each boar were pooled, distributed into 21 aliquots of 8-mL each, and centrifuged at 600× *g* and 16 °C for 5 min. Sperm pellet was immediately resuspended in capacitation medium (20 mM of HEPES, 112 mM of NaCl, 3.1 mM of KCl, 5 mM of glucose, 21.7 mM of sodium-L-lactate, 1 mM of sodium pyruvate, 0.3 mM of Na_2_HPO_4_, 0.4 mM of MgSO_4_·7H_2_O, 4.5 mM of CaCl_2_·2H_2_O, 5 mg/mL of bovine serum albumin (BSA), and 15 mM of bicarbonate) to a final concentration of 1 × 10^7^ spermatozoa/mL. Seven aliquots were maintained as controls (control samples). All samples were incubated at 38.5 °C, 100% humidity and 5% CO_2_ in a Hera Cell 150 incubator (Heraeus, Hanau, Germany) for 60 min (t1), 120 min (t2), 180 min (t3), 240 min (t4), 250 min (t4 + 10), 270 min (t4 h + 30) or 300 min (t5). Just before starting the experiment (t0), 100 nM of PAX were added to the capacitation medium in seven aliquots (PAX samples), and 20 mM of TEA in the other seven aliquots (TEA samples). PAX and TEA samples were incubated together with controls at the same conditions and for the same incubation times. Moreover, in those samples incubated for 250 (t4 + 10), 270 (t4 + 30) and 300 min (t5), 10 µg/mL of progesterone were added at 240 min (t4).

In the second experiment, seminal doses were divided into 13 aliquots of 8 mL each, centrifuged at 600× *g* and 16 °C for 5 min and resuspended in capacitation medium to a final concentration of 1 × 10^7^ spermatozoa/mL. Four aliquots were incubated at 38.5 °C and 5% CO_2_ for 60 min (t1), 120 min (t2), 180 min (t3) and 240 min (t4), whereas the other nine aliquots were incubated for 250 min (t4 + 10), 270 min (t4 + 30) or 300 min (t5). From these nine aliquots, three were maintained as controls and were added with 10 µg/mL of progesterone after 240 min of incubation (control samples). Three aliquots were added with 10 µg/mL of progesterone and 100 nM of PAX after 240 min of incubation (PAX acute samples). The remaining three aliquots were added with 10 µg/mL of progesterone and 20 mM of TEA (TEA acute samples) after 240 min of incubation.

At each relevant time point, i.e., 0 min (t0), 60 min (t1), 120 min (t2), 180 min (t3), 240 min (t4), 250 min (t4 + 10), 270 min (t4 h + 30) or 300 min (t5), the following sperm parameters were evaluated: sperm motility and kinematics using a CASA system; membrane lipid disorder, integrities of plasma and acrosome membranes and intracellular calcium levels by flow cytometry; and acrosin activity using a spectrophotometric assay.

The concentration of each inhibitor was chosen according to previously reported data and preliminary experiments. PAX at 100 nM blocks efficiently and specifically SLO1 channels in human spermatozoa [2]. TEA acts as an efficient blocker of all types of K^+^ channels when added to the cell medium at 10–20 mM [21,43].

### 4.4. Immunoblotting

For total protein extraction, sperm pellets and tissue samples (testicle, epididymis, ovarian and oviduct) were resuspended in RIPA lysis buffer (R0278), supplemented with 1:100 (*v*:*v*) protease inhibitor cocktail, 0.1 mM phenyl-methane-sulfonylfluoride (PMSF), and sodium orthovanadate 700 mM. After incubating in agitation at 4 °C for 30 min, samples were sonicated on ice three times (five pulses each; 20 KHz) every 2 min. Following this, samples were centrifuged at 10,000× *g* and 4 °C for 15 min and supernatants were collected. Subsequently, quantification of total protein was carried out in triplicate by a detergent compatible (DC) method (BioRad, Hercules, CA, USA), and samples were diluted at 1 μg of total protein per μL in lysis buffer.

Subsequently, 10 μL of each sample was mixed with 10 μL of Laemmli reducing buffer 1× containing 5% (*v*/*v*) β-mercaptoethanol (BioRad). Samples were boiled at 90 °C for 5 min and loaded onto Mini-PROTEAN^®^ TGX™ Precast Gels (BioRad). Electrophoresis was run at 200 V for 60 min (IEF Cell Protean System, BioRad). Stain-Free method is based on the fluorescent detection of tryptophan residues contained in the protein sequence which are previously modified by a trihalo compound included in the electrophoresis gel. Consequently, total protein bands were visualized by 2.5 min of UV exposition and subsequently acquisition with G:BOX Chemi XL 1.4 (SynGene, Frederick, USA). Proteins from gels were subsequently transferred onto polyvinylidene fluoride membranes using Trans-Blot^®^ Turbo™ (BioRad).

Membranes were blocked at room temperature under agitation for 1 h with TBS1× solution containing 10 mM of Tris (Panreac, Barcelona, Spain), 150 mM of NaCl (LabKem, Barcelona, Spain), 0.05% (*w*:*v*) Tween-20 (pH adjusted to 7.3; Panreac, Barcelona, Spain) and 5% bovine serum albumin (BSA, Roche Diagnostics, S.L.; Basel, Switzerland). Thereafter, membranes were incubated with a primary, SLO1/Maxi Potassium Channel Alpha antibody (ref. GTX54874, GeneTex; Irvine, CA, USA), which was previously diluted in blocking solution at 1:15,000 (*v*:*v*) overnight at 4 °C under agitation. In the next step, membranes were rinsed three times with washing solution (TBS1×-Tween20). Following this, membranes were incubated at room temperature under agitation for 1 h with a secondary, anti-rabbit antibody conjugated with horseradish peroxidase (ref. P0448, Agilent, Santa Clara, USA) diluted at 1:30,000 (*v*:*v*) in blocking solution. Protein bands were visualized with a chemiluminescent substrate (ImmobilionTM Western Detection Reagents, Millipore, Darmstadt, Germany) and scanned with G:BOX Chemi XL 1.4 (SynGene). Next, membranes were stripped and blocked prior to incubation with an anti-alpha-tubulin antibody (ref. MABT205, Millipore; 1:100,000, *v*:*v*) at room temperature for 1 h. Finally, membranes were washed thrice and incubated with a secondary anti-mouse antibody (ref. P0260, Agilent; 1:200,000, *v*:*v*) at room temperature for 1 h. Subsequently, membranes were washed, visualized and scanned.

The specificity of primary antibodies was previously confirmed through peptide competition assays utilizing SLO1-immunizing peptide (GTX54874-PEP; GeneTex), 20 times in excess with regard to the primary antibody. Additionally, positive controls were performed by using tissue samples of testicles, epididymis, ovarian and oviduct.

### 4.5. Immunofluorescence

Sperm samples were diluted to a final concentration of 3 × 10^6^ cells/mL and washed with PBS 1× at 500× *g* and room temperature for 5 min. Following this, sperm were fixed with 2% (*w*:*v*) paraformaldehyde at room temperature for 30 min. Then, samples were centrifuged twice at 500× *g* at room temperature for 5 min and resuspended with PBS1×. For each boar, 150 μL of sperm sample was placed onto ethanol-rinsed slides and incubated for at room temperature 1 h to promote sperm adhesion. To block non-specific binding sites and to permeabilize sperm cells, slides were treated with a blocking solution consisting of TBS-Tween20 1X containing 0.25% (*v*:*v*) Triton X-100 and 3% (w:v) BSA at room temperature for 1 h. Permeabilized spermatozoa were first incubated with the primary anti-SLO1 antibody (diluted at 1:100, *v*:*v*) at 4 °C overnight, and then with a secondary anti-rabbit conjugated with Alexa Fluor488 diluted 1:200 (*v*:*v*) in blocking solution at room temperature for 1 h. Following, samples were washed five times with PBS1× for 5 min. Finally, samples were mounted with 10 μL of Vectashield mounting medium containing 4,6-diamidino-2-phenylindole (DAPI; Vectorlabs, Burlingame, CA, USA). Spermatozoa were evaluated under a confocal microscope (CLSM Nikon A1R; Nikon Corp, Tokyo, Japan). Samples were excited at 405 nm in order to localize the DAPI-stained nucleus, whereas excitation at 496 nm was used to determine the localization of SLO1. Moreover, phase images were collected with a transmitted light detector. Three replicates of 100 spermatozoa each were counted. In negative controls, incubation with primary antibody was omitted. Finally, the specificity of primary antibodies was confirmed by separate peptide competition assays. Samples were incubated with SLO1 antibody and its corresponding immunizing peptide, which was 20 times in excess with regard to the primary antibody.

### 4.6. Evaluation of Sperm Motility and Kinematics

To measure sperm motility and kinematics, a 5-µL droplet was mounted onto a pre-warmed (38 °C) Makler chamber (Sefi-medical Instruments, Haifa, Israel). Sperm motility was examined at 100× magnification using a negative phase-contrast objective coupled to an Olympus BX41 microscope (Olympus Europe GmbH, Hamburg, Germany), and equipped with ISAS software (ISAS Ver. 1.0; Proiser S.L., Valencia, Spain). In all samples (control, PAX, TEA, PAX acute and TEA acute), three replicates of at least 1000 spermatozoa per replicate were examined. Twenty-five consecutive digitalized frames per second were acquired in each field, and total and progressive sperm motilities were assessed. A spermatozoon was considered to be motile when its VAP (velocity of the average pathway) was equal or higher than 10 µm/s, and progressively motile when its percentage of straightness (STR) was equal or higher than 45% [33]. For each incubation time and treatment, sperm motility was expressed as the percentage of total motile spermatozoa (TMOT) and the percentage of progressive motile spermatozoa (PMOT) (Figure 4) (mean ± SEM; *n* = 7).

In addition to the total and progressive sperm motilities, the following sperm kinematic parameters were also evaluated: curvilinear velocity (VCL, average velocity measured over the actual point-to-point track followed by the sperm head; µm/s), straight-line velocity (VSL, average path velocity of the sperm head along a straight line from its first to its last position; µm/s) and average path velocity (VAP, velocity of the sperm head along its average trajectory; µm/s). Indexes of linearity (LIN: ratio between VSL and VCL; %), straightness (STR: ratio between VSL and VAP; %) and oscillation (WOB: ratio between VAP and VCL; %) were also included. For each incubation time and treatment, the amplitude of lateral head displacement (ALH: average value of the extreme side-to-side movement of the sperm head in each beat cycle; µm), the beat cross frequency (BCF: frequency with which the actual sperm trajectory crosses the average path trajectory; Hz) was also recorded. Finally, hyperactive spermatozoa were those that showed ALH > 2.5 µm and STR > 85%, according with the parameters established by the ISAS software; Figure 4). Results are expressed as mean ± SEM (*n* = 7) (Supplementary Table S1).

### 4.7. Flow Cytometry Analyses

Flow cytometry was used to evaluate lipid disorder of plasma membrane, integrities of plasma and acrosome membranes, and intracellular calcium levels. In each assay, the sperm concentration was first adjusted to 1 × 10^6^ spermatozoa/mL in Beltsville Thawing Solution (BTS) in a final volume of 0.5 mL. Three replicates per incubation time, treatment and sperm parameter were examined in a Cell Laboratory QuantaSC™ cytometer (Beckman Coulter, Fullerton, CA, USA).

Information about flow cytometry analyses conducted in this work is given according to the recommendations of the International Society for Advancement of Cytometry (ISAC). Samples were excited with an argon ion laser (488 nm) set at a power of 22 mW. For each particle, characteristics were evaluated and plotted as Electronic Volume (EV, equivalent to Forward Scatter) and Side Scatter (SS). Three optical filters were used with the following optical properties: FL1 (green fluorescence): Dichroic/Splitter, DRLP: 550 nm, BP filter: 525 nm, detection width 505–545 nm, and FL3 (red fluorescence): LP filter: 670, detection width: 670 ± 30 nm). Sheath fluid flow rate was set at 4.17 μL/min in all analyses and a minimum of 10,000 events per replicate was evaluated. The analyzer threshold was adjusted on the EV channel to exclude subcellular debris (particles diameter <7 μm) and cell aggregates (particles diameter > 12 μm). The sperm-specific events were positively gated on the basis of EV/SS distributions. The other events were gated out.

In all flow cytometry assays, percentages of non-DNA-containing particles (alien particles) were determined to avoid an overestimation of sperm-events in the first quadrant (*q*1) [44]. Briefly, at each relevant time point, 5 µL of sperm sample were diluted in 895 µL of milliQ®-distilled water. Samples were subsequently stained with Propidium Iodide (PI) at a final concentration of 12 µM and incubated at 37.5 °C for 3 min. Percentages of alien particles (*f*) were used to correct the percentages of non-stained spermatozoa (*q*_1_) in each sample and staining protocol, according to the following formula:

$$q_{1}^{\prime }\;=\;\frac{q_{1}-f}{100-f}\;\times \;100$$

where *q*_1_’ is the percentage of non-stained spermatozoa after correction.

### 4.8. Plasma Membrane and Acrosome Membrane Integrity (PNA-FITC/PI)

Acrosome integrity was evaluated using the lectin from *Arachis hypogaea* (peanut agglutinin, PNA) conjugated with fluorescein isothiocyanate (FITC) and ethidium homodimer (3,8-diamino-5-ethyl-6-phenylphenanthridinium bromide; EthD-1), as described in [45]. In brief, samples were incubated with EthD-1 (final concentration: 2.5 µg/mL) at 37.5 °C for 5 min in the dark. Thereafter, samples were centrifuged at 2000× *g* and 16 °C for 30 s and then resuspended with PBS containing 4 mg/mL bovine serum albumin (BSA). Following this, samples were again centrifuged at the aforementioned conditions and then fixed and permeabilized by adding 100 µL of ice-cold methanol (100%) for 30 s. Methanol was removed by centrifugation at 2000× *g* and 16 °C for 30 s and pellets were resuspended with 250 µL PBS. Next, 0.8 µL PNA-FITC (final concentration: 2.5 µM) was added and samples were incubated at 25 °C in the dark for 15 min. Finally, samples were washed twice with PBS at 2000× *g* for 30 s and finally resuspended in PBS. 

Following staining, samples were evaluated with the flow cytometer and the following four sperm populations were identified: (1) viable spermatozoa with an intact acrosome (PNA-FITC+/EthD-1-); (2) viable spermatozoa with an exocytosed acrosome (PNA-FITC-/EthD-1-); (3) non-viable spermatozoa with an intact acrosome (PNA-FITC+/EthD-1+); and (4) non-viable spermatozoa with an exocytosed acrosome (PNA-FITC-/EthD-1+). Fluorescence of EthD-1 was detected through FL3, whereas that of PNA-FITC was detected through FL1. Results are expressed as percentages based on the population of spermatozoa (EthD-1-) (mean ± SEM; *n* = 7) (Figure 5).

### 4.9. Plasma Membrane Lipid Disorder (M540/YO-PRO-1)

Membrane lipid disorder was determined using Merocyanine 540 (M540; Fluka, 63876) and YO-PRO^®^-1 (Y-3603; Molecular Probes, Invitrogen, ThermoFisher Scientific) [46,47]. In brief, sperm samples were stained with M540 (final concentration: 2.6 µM) and YO-PRO-1 (final concentration: 25 nM), and incubated at 37.5 °C for 10 min. YO-PRO-1 fluorescence was collected through FL1 filter, whereas M540 fluorescence was collected through FL3. The following sperm populations were identified [48,49]: (1) non-viable spermatozoa with low membrane lipid disorder (M540-/YO-PRO-1+); (2) non-viable spermatozoa with high membrane lipid disorder (M540+/YO-PRO-1-); (3) viable spermatozoa with low membrane lipid disorder (M540-/YO-PRO-1-); and (4) viable spermatozoa with high membrane lipid disorder (M540+/YO-PRO-1-). Results are expressed as percentages of viable spermatozoa with low membrane lipid disorder (M540-/YO-PRO-1-) and of viable spermatozoa with high membrane lipid disorder (M540+/YO-PRO-1-). Results are expressed as percentages based on the population of spermatozoa (YO-PRO-1-) (mean ± SEM; *n* = 7) (Figure 6). 

### 4.10. Determination of Intracellular Calcium Levels (Fluo3/PI and Rhod5/YO-PRO-1)

Intracellular calcium levels were evaluated with two separate markers (Fluo3-AM and Rhod5), which have been reported to have more affinity for the calcium stores of the mid-piece (Fluo3-AM) and of the sperm head (Rhod5), respectively [31]. In the first case, spermatozoa were stained with Fluo3-acetomethoxy ester fluorochrome (Fluo3-AM, F-1241; Molecular Probes, Invitrogen; final concentration: 1 µM) in combination with PI (final concentration: 12 µM). After the joint addition of the two fluorochromes, sperm samples were incubated at 37.5 °C for 10 min in darkness [50]. Fluo3-fluorescence was collected through FL1 and PI fluorescence by FL3. According to their fluorescence emission, viable (PI-) and non-viable (PI+) spermatozoa could show either low (Fluo3-) and high (Fluo3+) intracellular calcium levels. Results are expressed as the mean percentages of viable spermatozoa with low intracellular calcium levels (Fluo3-/PI-) and of viable spermatozoa with high intracellular calcium levels (Fluo3+/PI-) (mean ± SEM; *n* = 7). Results are expressed as percentages based on the population of spermatozoa (PI-) and as geometric fluorescence intensity of Fluo3 in viable spermatozoa with high intracellular calcium levels (Fluo3+/PI-) (mean ± SEM; *n* = 7; Figure 7). 

The other calcium marker (Rhod5-N, 2-(6-Amino-3-imino-3H-xanthen-9-yl) benzoic acid methyl ester; final concentration: 5 µM) was combined with YO-PRO-1 (final concentration: 25 nM). After the joint addition of both fluorochromes, the sperm samples were incubated at 37.5 °C for 10 min in darkness [33]. YO-PRO-1 fluorescence was collected through FL1 and Rhod5 fluorescence through FL3. Results are expressed as percentages of viable spermatozoa with low intracellular calcium levels (Rhod5-/YO-PRO-1-) and of viable spermatozoa with high intracellular calcium levels (Rhod5+/YO-PRO-1-). Results are expressed as percentages based on the population of spermatozoa (YO-PRO-1-) and as geometric fluorescence intensity of Rhod5 in viable spermatozoa with high intracellular calcium levels (Rhod5+/YO-PRO-1-) (mean ± SEM; *n* = 7; Figure 8).

### 4.11. Acrosin Activity Assay

For each incubation time and tretment, acrosin activity was determined spectrophotometrically following a previously described method [51], which was adapted in our laboratory [32]. For each sperm sample, 100 µL containing 5 × 10^6^ spermatozoa were centrifuged at 1000× *g* and 16 °C for 30 min over 500 µL of 11% Ficoll to separate spermatozoa from the medium. The sperm pellet was suspended in 1 mL of a buffer solution containing 0.01% Triton X-100 and 23 mM of N-α-benzoyl-DL-arginine l-nitroanilide hydrochloride (BAPNA) substrate. Hydrolyzed BAPNA by acrosin formed a chromoforic product, 4-nitroanilin, which was detected after 3 h of incubation at room temperature using a UV-1600PC VWRTM spectrophotometer (VWR; Leuven, Belgium; wavelength: 410 nm). For each sperm sample, total acrosin activity (µIU acrosina/10^6^ spermatozoa) was calculated using the formula described by Langlois et al. [48]. Three replicates per incubation time and sample treatment were evaluated and results are expressed as the mean ± SEM (*n* = 7) (Figure 9).

### 4.12. Statistical Analyses

Statistical analyses were performed using IBM SPSS 24.0 for Windows (IBM Corp., Armonk, NY, USA) and figures were drawn with Origin Pro 8.0 software (OriginLab Corp., Northampton, MA, USA).

Sperm quality and function parameters (sperm motility, sperm kinematics, integrities of plasma and acrosome membranes, membrane lipid disorder, intracellular calcium levels and acrosin activity) were considered as dependent variables, whereas each experiment and incubation treatments using seminal samples from different boars (*n* = 7) were treated as biological replicates. All the variables were first tested for normality (Shapiro-Wilk test) and homoscedasticity (Levene test). Data were then evaluated with a mixed model (intra-subject factor: incubation time; inter-subject factor: treatment) followed by the post-hoc Sidak test for pair-wise comparisons.

In all the statistical analyses, the significant level was set at *p* ≤ 0.05. Results are expressed as means ± standard error of the mean (SEM) (*n* = 7).

## 5. Conclusions

In conclusion, our results provide compelling evidence that not only Ca^2+^, but also K^+^ conductance, are important in triggering sperm capacitation in boar spermatozoa. This study also demonstrates, for the first time, the presence of SLO1 channels in the flagellum and the anterior post-acrosomal region of boar spermatozoa. While the pharmacological approach suggests that SLO1 channels are crucial for K^+^ conductance in the sperm head during in vitro capacitation of boar spermatozoa and appear to be involved in the acrosome exocytosis induced by progesterone, they do not seem to be crucial for K^+^ conductance throughout the sperm tail. Therefore, further research is needed in order to identify other K^+^ channels present in the flagellum of boar spermatozoa.

## Supplementary Materials

Supplementary Materials can be found at https://www.mdpi.com/1422-0067/20/24/6330/s1.

## Abbreviations

ALH	Amplitude of lateral head displacement
BCF	Beat cross frequency
BK	Big potassium channel
EV	Electronic volume
FITC	Fluorescein isothiocyanate
HVCN1	Voltage-gated H^+^ channel 1
ISAC	International Society for Advancement of Cytometry
LIN	Linearity
M540	Merocyanine 540
PAX	Paxilline
PI	Propidium iodide
PMOT	Progressive motility
PNA	Peanut agglutinin
SEM	Standard error of the mean
SS	Side scatter
STR	Straightness
TEA	Tetraethyl ammonium
TMOT	Total motility
VAP	Average path velocity
VCL	Curvilinear velocity
VSL	Straight line velocity
