# Supplementary material for: Elucidating the Role of K+ Channels during In Vitro Capacitation of Boar Spermatozoa: Do SLO1 Channels Play a Crucial Role?
Source: Int J Mol Sci. 2019 Dec 15;20(24):6330. doi: 10.3390/ijms20246330 (PMC6940911; doi:10.3390/ijms20246330)
Supplement: Supplementary file 1 [file ijms-20-06330-s001.pdf]

Supplementary Table 1. Sperm kinematics during *in vitro* capacitation of boar spermatozoa in a capacitation medium either supplemented or not with PAX or TEA inhibitors either at 0 min (Experiment 1, PAX and TEA media) or 240 min of incubation (Experiment 2, PAX acute or TEA acute media).

Kinematic parameter	Incubation time	Experiment 1			Experiment 2		
		Capacitation medium	PAX medium	TEA medium	Capacitation medium	PAX acute medium	TEA acute medium
VCL	0	51.05 ± 3.48[a]	51.05 ± 3.48[a]	51.05 ± 3.48[a]	54.38 ± 5.02[a]		
	60	75.23 ± 4.23[b,1]	74.15 ± 4.24[b,1,2]	67.02 ± 2.94[b,2]	80.02 ± 5.94[b]		
	120	64.31 ± 3.79[c,1]	69.54 ± 4.01[b,c,1]	56.83 ± 3.86[c,2]	74.19 ± 5.85[b,c]		
	180	64.41 ± 4.24[c,1]	64.50 ± 2.48[c,d,1]	51.73 ± 4.93[c,2]	69.86 ± 5.80[c]		
	240	58.03 ± 5.55[c,1]	61.94 ± 5.17[c,d,1]	42.48 ± 4.46[d,2]	65.29 ± 5.36[c]		
	250	58.55 ± 4.38[c,1]	63.36 ± 4.17[c,d,1]	41.03 ± 4.64[d,2]	76.77 ± 6.91[b,c,1]	72.96 ± 4.91[b,c,1]	54.37 ± 5.63[d,2]
	270	62.20 ± 4.70[c,1]	64.48 ± 5.69[c,d,1]	38.69 ± 3.86[d,2]	76.93 ± 5.41[b,c,1]	71.92 ± 1.88[c,1]	55.36 ± 5.11[d,2]
	300	62.68 ± 5.45[c,1]	60.57 ± 4.53[d,1]	30.18 ± 3.42[e,2]	82.94 ± 6.14[b,1]	80.35 ± 3.49[b,1]	58.23 ± 5.25[a,c,d,2]
VSL	0	30.71 ± 2.87[a]	30.71 ± 2.87[a,c]	30.71 ± 2.87[a,c]	31.69 ± 3.75[a,b]		
	60	38.12 ± 2.30[b,1]	37.83 ± 2.76[b,1]	36.75 ± 2.92[b,1]	33.51 ± 2.12[a]		
	120	29.46 ± 2.51[a,1]	35.97 ± 3.05[a,b,2]	30.04 ± 2.90[a,1,2]	26.98 ± 3.06[b]		
	180	27.04 ± 2.69[a,1,2]	29.01 ± 2.46[c,2]	23.69 ± 2.60[c,1]	24.35 ± 3.06[b]		
	240	25.33 ± 3.39[a,1,2]	27.84 ± 2.37[c,1]	19.60 ± 2.61[c,d,2]	25.51 ± 3.36[b]		
	250	26.13 ± 3.08[a,1]	30.04 ± 3.16[b,c,1]	19.32 ± 2.68[c,d,2]	24.29 ± 3.46[b,1]	24.35 ± 3.49[b,1]	17.33 ± 2.49[c,2]
	270	31.83 ± 3.34[a,1]	34.06 ± 3.52[a,b,1]	16.47 ± 2.36[d,e,2]	23.15 ± 3.65[b,1]	29.55 ± 2.18[a,b,2]	21.08 ± 2.12[b,c,1]
	300	29.22 ± 3.20[a,1]	27.57 ± 2.80[c,1]	11.86 ± 2.70[e,2]	36.68 ± 3.04[a,1]	35.02 ± 3.03[a,1]	24.87 ± 2.58[b,2]
VAP	0	39.61 ± 3.40[a]	39.61 ± 3.40[a]	39.61 ± 3.40[a,c]	42.24 ± 4.51[a,b]		
	60	52.39 ± 2.91[b,1]	52.01 ± 3.41[b,1]	47.29 ± 2.84[b,1]	50.80 ± 3.98[a]		
	120	42.21 ± 2.71[a,1]	48.49 ± 3.04[b,2]	39.74 ± 3.09[a,1]	45.67 ± 3.99[a,b]		
	180	38.91 ± 3.08[a,1]	40.74 ± 2.18[a,1]	32.67 ± 2.85[c,2]	39.34 ± 3.53[b]		
	240	36.45 ± 3.65[a,1]	39.13 ± 4.05[a,1]	26.59 ± 2.48[d,2]	38.08 ± 3.61[b]		
	250	37.04 ± 3.26[a,1]	41.49 ± 3.76[a,1]	26.66 ± 2.21[d,2]	42.44 ± 3.90[a,b,1]	39.27 ± 2.94[b,1]	29.70 ± 2.25[c,2]
	270	42.52 ± 3.83[a,1]	44.69 ± 3.92[a,1]	23.95 ± 2.95[d,2]	39.19 ± 2.77[b,1]	44.70 ± 2.91[a,b,1]	32.11 ± 2.15[c,d,2]

	300	40.64 ± 3.40[a,1]	38.54 ± 3.00[a,1]	18.00 ± 2.48[e,2]	50.88 ± 3.99[a,1]	49.87 ± 2.04[a,1]	35.66 ± 2.75[d,2]
LIN	0	59.32 ± 2.02[a]	59.32 ± 2.02[a]	59.32 ± 2.02[a]	57.89 ± 1.82[a]		
VSL/VCL	60	51.92 ± 3.70[b,1]	51.73 ± 3.63[b,1]	55.07 ± 3.80[a,b,1]	42.25 ± 1.73[b]		
	120	46.87 ± 4.33[b,c,1]	52.28 ± 3.95[b,1]	52.76 ± 3.03[b,c,1]	36.53 ± 4.82[b,c]		
	180	42.73 ± 4.01[c,1]	44.66 ± 4.02[b,1]	44.50 ± 4.75[c,d,1]	33.26 ± 2.38[c]		
	240	43.12 ± 4.09[c,1]	43.73 ± 4.41[b,1]	42.65 ± 4.76[d,1]	36.90 ± 4.76[b,c]		
	250	43.35 ± 4.61[c,1]	46.20 ± 4.80[b,1]	44.90 ± 3.93[c,d,1]	29.99 ± 3.15[c,1]	32.65 ± 4.72[b,1]	30.46 ± 3.86[b,1]
	270	50.36 ± 4.17[b,c,1]	50.72 ± 4.72[b,1]	42.39 ± 4.06[d,1]	33.77 ± 1.12[c,1]	40.47 ± 2.31[c,2]	37.73 ± 3.93[b,c,1,2]
	300	45.04 ± 4.60[b,c,1]	43.32 ± 4.85[b,1]	37.06 ± 3.22[d,1]	43.62 ± 4.84[b,1]	43.99 ± 4.34[c,1]	42.05 ± 3.99[c,1]
STR	0	76.93 ± 1.35[a]	76.93 ± 1.35[a]	76.93 ± 1.35[a]	74.36 ± 1.76[a]		
VSL/VAP	60	72.55 ± 3.45[a,b,1]	72.94 ± 3.34[a,b,1]	76.89 ± 2.82[a,1]	66.26 ± 3.09[b]		
	120	69.04 ± 3.56[b,1]	73.81 ± 3.84[a,b,1]	74.00 ± 2.80[a,b,1]	59.18 ± 3.48[c]		
	180	68.23 ± 3.10[b,1]	68.99 ± 3.81[b,1]	69.09 ± 4.19[b,c,1]	59.77 ± 2.58[c]		
	240	66.99 ± 3.83[b,1]	68.24 ± 4.49[b,1]	67.21 ± 4.05[b,c,1]	65.89 ± 3.38[b]		
	250	66.81 ± 4.93[b,1]	67.82 ± 4.25[b,1]	69.54 ± 3.47[b,c,1]	54.48 ± 4.02[c,1]	59.20 ± 4.03[c,1]	56.08 ± 4.24[c,1]
	270	72.98 ± 2.74[a,b,1]	72.04 ± 4.19[a,b,1]	64.74 ± 4.02[c,2]	58.69 ± 2.06[c,1]	66.11 ± 2.51[b,2]	64.69 ± 4.12[b,2]
	300	67.99 ± 4.46[b,1]	66.27 ± 4.37[b,1]	60.21 ± 4.22[c,1]	69.17 ± 4.33[b,1]	69.38 ± 4.12[b,1]	69.44 ± 2.28[b,1]
WOB	0	76.71 ± 1.63[a]	76.71 ± 1.63[a]	76.71 ± 1.63[a]	77.43 ± 1.38[a]		
VAP/VCL	60	70.18 ± 2.49[b,1]	70.32 ± 2.33[b,1]	70.60 ± 2.61[b,1]	63.61 ± 0.49[b]		
	120	66.27 ± 3.22[c,1]	70.03 ± 2.38[b,1]	69.79 ± 1.66[b,1]	61.36 ± 2.84[b,c]		
	180	61.07 ± 3.66[c,1]	63.11 ± 2.80[c,1]	62.31 ± 3.44[c,1]	55.11 ± 3.26[c]		
	240	62.81 ± 2.76[c,1]	61.71 ± 3.32[c,1]	60.57 ± 3.08[c,1]	55.25 ± 3.51[c]		
	250	62.87 ± 3.38[c,1]	65.04 ± 3.98[c,1]	63.08 ± 3.25[c,1]	53.80 ± 4.87[c,1]	53.12 ± 4.20[c,1]	53.71 ± 2.74[c,1]
	270	67.66 ± 3.60[c,1]	67.89 ± 3.55[c,1]	61.40 ± 3.66[c,1]	57.53 ± 3.38[c,1]	61.57 ± 2.72[b,1]	58.02 ± 2.67[c,1]
	300	63.86 ± 3.61[c,1]	62.58 ± 3.56[c,1]	58.38 ± 3.49[c,1]	60.96 ± 4.56[b,c,1]	62.24 ± 3.93[b,1]	60.30 ± 4.64[b,c,1]
ALH	0	2.01 ± 0.07[a]	2.01 ± 0.07[a]	2.01 ± 0.07[a]	2.02 ± 0.22[a]		
	60	2.72 ± 0.14[b,1]	2.72 ± 0.13[b,1]	2.42 ± 0.09[b,2]	2.98 ± 0.20[b,c]		
	120	2.29 ± 0.14[c,1]	2.54 ± 0.14[b,c,1]	2.04 ± 0.16[a,2]	2.48 ± 0.29[b]		
	180	2.43 ± 0.13[c,1]	2.35 ± 0.14[c,d,1]	1.84 ± 0.18[a,c,2]	2.42 ± 0.38[b]		
	240	2.48 ± 0.20[c,1]	2.40 ± 0.20[c,d,1]	1.57 ± 0.26[c,d,2]	2.54 ± 0.38[b,c]		
	250	2.18 ± 0.19[a,1]	2.22 ± 0.22[a,c,d,1]	1.53 ± 0.21[c,d,2]	2.65 ± 0.24[b,c,1]	2.66 ± 0.27[b,1]	1.95 ± 0.36[b,2]

	270	2.28 ± 0.18^{a,1}	2.24 ± 0.24^{a,c,d,1}	1.40 ± 0.21^{c,d,2}	2.56 ± 0.25^{b,c,1}	2.55 ± 0.05^{b,1}	2.01 ± 0.26^{b,2}
	300	2.16 ± 0.21^{a,1}	2.17 ± 0.27^{a,c,d,1}	0.99 ± 0.23^{d,2}	2.98 ± 0.10^{c,1}	2.94 ± 0.11^{c,1}	1.97 ± 0.32^{b,2}
BCF	0	7.32 ± 0.24^{a}	7.32 ± 0.24^{a}	7.32 ± 0.24^{a}	7.14 ± 0.56^{a}		
	60	8.04 ± 0.32^{b,1}	8.07 ± 0.25^{b,1}	7.79 ± 0.46^{a,1}	7.96 ± 0.43^{a}		
	120	7.24 ± 0.40^{a,1}	7.78 ± 0.37^{b,c,1}	7.02 ± 0.42^{a,1}	7.05 ± 0.69^{a,c}		
	180	7.07 ± 0.31^{a,c,1}	6.71 ± 0.48^{c,d,1}	5.76 ± 0.49^{b,2}	5.56 ± 0.86^{b,c}		
	240	6.41 ± 0.36^{c,d,1}	6.31 ± 0.36^{d,1}	5.22 ± 0.41^{b,2}	6.21 ± 0.82^{c}		
	250	6.94 ± 0.34^{a,d,1}	6.51 ± 0.39^{d,1}	4.85 ± 0.41^{b,2}	5.66 ± 0.96^{b,c,1}	6.28 ± 0.62^{c,1}	5.16 ± 0.57^{c,1}
	270	6.48 ± 0.32^{c,d,1}	6.69 ± 0.42^{d,1}	3.94 ± 0.30^{c,2}	5.89 ± 0.53^{b,c,1}	6.95 ± 0.27^{c,2}	5.59 ± 0.39^{c,1}
	300	6.24 ± 0.41^{d,1}	5.92 ± 0.47^{d,1}	3.10 ± 0.42^{c,2}	7.47 ± 0.79^{a,c,1}	7.30 ± 0.43^{c,1}	5.18 ± 0.59^{c,2}

Results are expressed as the mean ± error standard (*n*=**10**).

$^{a-e}$ superscripts indicate significant differences among incubation times within the same incubation medium.

$^{1-3}$ superscripts indicate significant differences among incubation media within the same incubation time and experiment.
